# Comparison of the Whole Cell Proteome and Secretome of Epidemic *Bordetella pertussis* Strains From the 2008–2012 Australian Epidemic Under Sulfate-Modulating Conditions

**DOI:** 10.3389/fmicb.2018.02851

**Published:** 2018-11-27

**Authors:** Laurence Don Wai Luu, Sophie Octavia, Ling Zhong, Mark J. Raftery, Vitali Sintchenko, Ruiting Lan

**Affiliations:** ^1^School of Biotechnology and Biomolecular Sciences, University of New South Wales, Sydney, NSW, Australia; ^2^Bioanalytical Mass Spectrometry Facility, University of New South Wales, Sydney, NSW, Australia; ^3^Centre for Infectious Diseases and Microbiology–Public Health, Institute of Clinical Pathology and Medical Research – NSW Health Pathology, Westmead Hospital, Sydney, NSW, Australia; ^4^Marie Bashir Institute for Infectious Diseases and Biosecurity, Sydney Medical School, University of Sydney, Sydney, NSW, Australia

**Keywords:** acellular vaccine, *Bordetella pertussis*, iTRAQ, proteomics, secretome, MRM-hr, sulfates, gene expression profiling

## Abstract

Sulfate is an important modulator for virulence factor expression in *Bordetella pertussis*, the causative organism for whooping cough. During infection, sulfate is released when respiratory epithelial cells are damaged which can affect gene expression. The current predominant strains in Australia are found in single nucleotide polymorphism (SNP) cluster I (*ptxP3/prn2*). It has been reported that *ptxP3* strains have higher mRNA expression of virulence genes than *ptxP1* strains under intermediate sulfate-modulating conditions (5 mM MgSO_4_). Our previous proteomic study compared L1423 (cluster I, *ptxP3*) and L1191 (cluster II, *ptxP1*) in Thalen–IJssel (THIJS) media without sulfate modulation and identified an upregulation of transport proteins and a downregulation of immunogenic proteins. To determine whether proteomic differences exist between cluster I and cluster II strains in intermediate modulating conditions, this study compared the whole cell proteome and secretome between L1423 and L1191 grown in THIJS media with 5 mM MgSO_4_ using iTRAQ and high-resolution multiple reaction monitoring (MRM-hr). Two proteins (BP0200 and BP1175) in the whole cell were upregulated in L1423 [fold change (FC) >1.2, false discovery rate (FDR) <0.05]. In the secretome, four proteins from the type III secretion system (T3SS) effectors were downregulated (FC < 0.8, FDR < 0.05) while six proteins, including two adhesins, pertactin (Prn) and tracheal colonization factor A (TcfA), were upregulated which were consistent with our previous proteomic study. The upregulation of Prn and TcfA in SNP cluster I may result in improved adhesion while the downregulation of the T3SS and other immunogenic proteins may reduce immune recognition, which may contribute to the increased fitness of cluster I *B. pertussis* strains.

## Introduction

*Bordetella pertussis* is the causative agent for whooping cough, a re-emerging vaccine preventable disease that disproportionately affects infants. The current vaccine used in Australia is the acellular vaccine (ACV) which replaced the previous whole cell vaccine (WCV) in 2000 (Royle and Lambert, 2015). The three-component ACV is the main vaccine used in Australia and contains three virulence factors: pertussis toxin (Ptx), pertactin (Prn), and filamentous hemagglutinin (FHA). In addition to these virulence factors, *B. pertussis* also produces other virulence factors such as the type III secretion system (T3SS), several toxins [adenylate cyclase toxin (CyaA), tracheal cytotoxin, and dermonecrotic toxin] and several autotransporters involved in adhesion and serum resistance [tracheal colonization factor (TcfA), BipA, serum resistance protein (BrkA), and Vag8] (Melvin et al., 2014).

The expression of these virulence genes is regulated by the BvgAS, a two-component system governing a regulon in response to changes in environmental signals. This regulator responds to changes in environmental stimuli including temperature and the presence of modulators such as sulfate and nicotinic acid (Lacey, 1960; Prugnola et al., 1995; Nakamura et al., 2006). BvgS consists of two Venus flytrap (VFT) periplasmic domains, followed by a Per-Arnt-Sim (PAS) domain and a cytoplasmic histidine kinase (HK) moiety. Two α-helices, linkers 1 and 2, connect the VFT to the PAS domain and HK. BvgS kinase activity is constitutively active under basal conditions when linker 1 adopts a coiled-coil conformation. However, when sulfate or other negative modulators are present, it disrupts the coiled coil conformation of linker 1 which affects the PAS domain interface and causes linker 2 to adopt the coiled coil position resulting in the phosphatase mode (Dupré et al., 2013, 2015; Lesne et al., 2016, 2017, 2018).

Under non-modulating (Bvg+) conditions (37°C and no modulators), BvgS is autophosphorylated. This phosphorylation is then passed through a series of histidine–aspartate transporters and eventually to BvgA. Phosphorylated BvgA will bind to virulence gene promoters and promote transcription (Scarlato et al., 1990). However, when the temperature is 26°C or modulators (sulfate or nicotinic acid) are present, BvgS and BvgA are not phosphorylated (Bvg-), leading to the repression of virulence genes (Scarlato et al., 1990, 1991). A third phenotype, Bvgi, occurs when there are intermediate modulating conditions which result in lowered levels of phosphorylated BvgA, thereby promoting transcription of a subgroup of virulence genes (Vergara-Irigaray et al., 2005). Different promoters have different affinity for phosphorylated BvgA based on the number of binding sites present. This affects their expression temporally and response to modulators with genes such as *fhaB* and *fim* expressed early during Bvg activation while *ptx* and *cyaA* are expressed later and are more sensitive to modulation (Scarlato et al., 1991; Kinnear et al., 2001; Veal-Carr and Stibitz, 2005). Bvg+ is necessary and sufficient for infection (Smith et al., 2001), while Bvgi can be important for survival during transmission (Vergara-Irigaray et al., 2005). The role of Bvg- in *B. pertussis* infection is currently unknown. Bvg- phase-locked mutants were shown to be avirulent (Merkel et al., 1998); however, recent studies suggest that the Bvg- phase may be involved in survival, transmission, and persistence (Hot et al., 2003; Coutte et al., 2016; Moon et al., 2017; van Beek et al., 2018).

Several factors have been linked with the resurgence of pertussis. These factors include improved diagnostic methods, differences in relative vaccine effectiveness, reduced vaccination coverage levels, waning immunity, differences in vaccine immune response, and pathogen adaptation to ACVs (Mooi et al., 2014). It is likely that resurgence is caused by a combination of these factors and the extent of each factor’s influence on resurgence is likely to differ in different countries. Despite this, two of the main factors attributed to the increase of pertussis in many countries with high vaccination coverage are the ability of the ACV to protect against disease but not against transmission (Warfel et al., 2014) and pathogen adaptation to the ACV through antigenic mismatches and differences in gene expression (Mooi et al., 2014). It has been shown that ACV immunization elicits predominantly a Th2 response which protects against pertussis disease but does not prevent colonization and transmission of the bacteria to unvaccinated individuals in a baboon model (Ryan et al., 1998; Warfel et al., 2014). Single nucleotide polymorphism (SNP) typing of circulating strains isolated after the introduction of ACVs, found that they primarily belonged to SNP cluster I and contains the alleles *ptx* promoter 3 *(ptxP3)* and *prn2* (Octavia et al., 2011, 2012). SNP cluster I strains have replaced the previously predominant SNP cluster II strains which contained *ptxP1/prn3* alleles, and they were responsible for the 2008–2012 Australian epidemic. Notably, *ptxP3* strains appeared to produce larger amounts of Ptx (Mooi et al., 2009).

A mixed-infection mice model using representative strains from SNP cluster I (L1423) and II (L1191) showed that cluster I strains were fitter than cluster II in both three-component ACV vaccinated and unvaccinated hosts (Safarchi et al., 2016a). Previous transcriptomic studies have documented differences in the expression of virulence and non-virulence genes between *ptxP3* and *ptxP1* strains (King et al., 2013). However, our previous comparative global proteomics study between the cluster I and II representative strains showed that there were no differences in the expression of ACV antigens but identified other key protein differences including an increase in TcfA and several transport/binding proteins for phosphate, metals, and amino acids in SNP cluster I and a decrease in immunogenic proteins such as Bsp22, theT3SS tip protein, and lipoprotein BP1569, a toll-like receptor 2 agonist (Luu et al., 2018). Similarly, a study by de Gouw et al. (2014b) also found very little difference in gene expression between Dutch *ptxP3* and *ptxP1* strains under non-modulating conditions with nine genes significantly downregulated and eight upregulated. However when grown in the presence of intermediate level of sulfate (5 mM MgSO_4_), *ptxP3* strains were found to be less sensitive to sulfate modulation of Bvg and had increased mRNA expression of ACV antigens such as *ptx* and other virulence factors including *vag8* and T3SS. de Gouw et al. (2014b) suggested that during infection, the lysis of host respiratory epithelial cells leads to increased sulfate concentration, possibly through desulfation of host proteins. This results in Bvg modulation and repression of virulence genes (de Gouw et al., 2014b). Decreased sensitivity to modulation may allow *B. pertussis* to remain virulent for longer periods of time leading to increased transmission and infection (de Gouw et al., 2014b). We hypothesized that differential response to sulfate modulation of Australian SNP cluster I strains compared to SNP cluster II may also contribute to increased fitness observed *in vivo* (Safarchi et al., 2016a). Therefore, to determine whether additional expression differences exist between the two clusters under modulating conditions, the aim of this study was to use isobaric tag for relative and absolute quantitation (iTRAQ and MRM-hr to compare the global proteome of our representative SNP cluster I and II strain under intermediate sulfate-modulating condition (5 mM MgSO_4_).

## Materials and Methods

### Bacterial Growth Conditions

*Bordetella pertussis* strains L1423 (cluster I – *ptxP3*, *prn2*, *ptxA1*, and *fim3A*) and L1191 (cluster II – *ptxP1*, *prn3*, *ptxA1*, and *fim3A*) from glycerol stocks were grown on Bordet–Gengou (BG, BD scientific) media plates in parallel and incubated at 37°C for 3–5 days. Pure cultures were obtained by subculturing a single (small, hemolytic) Bvg+ colony onto a second BG plate and incubated again at 37°C. Pure Bvg+ (small, hemolytic) colonies were then inoculated into THIJS broth (Thalen et al., 1999) with 1% Heptakis [(2,6-*O*-dimethyl) β-cyclodextrin] and THIJS supplement, and incubated at 37°C with shaking. After 24 h, the cultures were centrifuged and the cell pellets were resuspended in 100 ml THIJS with 5 mM MgSO_4_ and a starting OD_600_ of 0.05. The cultures were incubated for 12 h and afterward, the CFU/ml were recorded. Six biological replicates were performed for each strain.

### RNA Extraction and RT-qPCR

One milliliter of culture was aliquoted from each sample with 5% v/v phenol in ethanol added to prevent RNA degradation (Bhagwat et al., 2003). RNA was extracted using the ISOLATE II RNA mini kit (Bioline) according to the manufacturer’s instructions and further treated with DNase I [New England Biolabs (NEB)] until all traces of genomic DNA were removed. cDNA synthesis was performed using the Tetro cDNA synthesis kit (Bioline). RT-qPCR was performed using the SensiFAST SYBR Hi-ROX kit (Bioline) on the Corbett Rotor-Gene 6000 (Qiagen) to quantify the relative expression of *bipA* against *recA*, the housekeeping gene (primers used listed in Supplementary Table S1) (Stefanelli et al., 2006). The ΔΔCt method (Schmittgen and Livak, 2008) was used to calculate the relative expression of *bipA* between strains grown with and without 5 mM MgSO_4_. Statistical significance was calculated using a two-sided Student’s *t*-test with *p* < 0.05 defined as significant.

### iTRAQ

**FIGURE 1:**
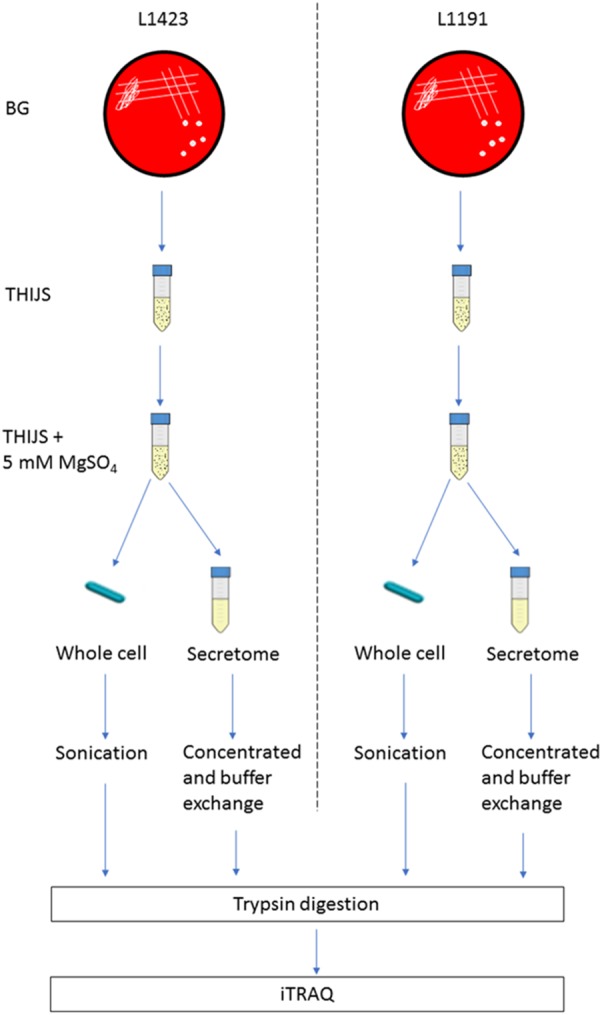
Experimental design of iTRAQ experiment. L1423 and L1191 were grown simultaneously in parallel on BG plates. Pure small, hemolytic Bvg+ colonies were then inoculated into THIJS and then into THIJS + 5 mM MgSO_4_. After 12 h incubation, the whole cell was sonicated and the secretome concentrated. Protein samples were then digested and labeled with iTRAQ reagents.

Whole cell and supernatant samples for iTRAQ and MRM-hr were prepared as previously described in (Luu et al., 2018; Figure 1). Briefly, centrifugation separated the whole cell and supernatant. The whole cell was then lysed via sonication and the supernatant (100 ml) was concentrated to 200–1000 μl using a 5-kDa Amicon filter (Millipore).

For iTRAQ, two seven-plex iTRAQs were performed for whole cell and supernatant as done previously. Each seven-plex iTRAQ contained three biological replicates from L1423, three biological replicates from L1191, and one pooled reference sample for normalization between iTRAQ experiments. One hundred micrograms of protein from each sample were reduced and alkylated, trypsin digested and labeled with isobaric tags (SCIEX). The labeled samples were then combined, and excess label and salt removed using a cation exchange cartridge followed by an Oasis cartridge clean-up. The combined labeled samples were analyzed using a TripleTOF-5600+ (SCIEX) coupled to a Dionex UltiMate 3000 RSLCnano pump system, Switchos valve unit, and Famos autosampler (Thermo Scientific Dionex). A replicate LC/MS run was performed for each iTRAQ sample.

Protein identification and quantification were performed using ProteinPilot v5.0 (Applied Biosystems) (Shilov et al., 2007) with a custom *B. pertussis* database. Proteins identifications were accepted with unused score >1.3 (>95% confidence) and >1 peptide. Quantification of proteins was performed using common proteins between both iTRAQs. FCs were calculated in relation to L1423 with L1191 as the reference. Upregulated proteins in L1423 were defined as having FCs >1.2 while downregulated proteins in L1423 had FCs <0.8. Significance was determined using a Student’s *t*-test with FDR correction (Storey and Tibshirani, 2003). *p* < 0.05 and *q* < 0.05 was the cutoff for significance. Virulence proteins that showed an upregulated or downregulated (FC > 1.2 or <0.8 and *q* < 0.1 and *q* < 0.2) trend were also assessed (Luu et al., 2018).

Cellular location and functional categories were assigned as previously described (Luu et al., 2017). Functional enrichment analysis of proteins was performed using T-profiler adapted to *B. pertussis* functional categories. T-profiler is a program which was developed for transcriptomic data to determine overall expression differences between predefined genes in functional groups using a *T*-test (Boorsma et al., 2005). Although originally developed for microarrays, T-profiler has since been applied to proteomics data in *Listeria monocytogenes* and *Escherichia coli* O157:H7 (Bowman et al., 2012; Kocharunchitt et al., 2014). *T*-value and *p*-value were obtained for each functional category using T-profiler. Briefly, for each predefined protein functional category according to Bart et al. (2010), a two-tailed *t*-test was performed for each functional category using the mean log2 FC of N proteins assigned to that category against the mean log2 FC of N proteins not assigned to that functional category. To decrease the effect of outliers, the highest and lowest log2 FC value in each gene group were removed prior to calculation as described by Boorsma et al. (2005). Functional categories with *T*-values >0 were defined as upregulated in cluster I while those with *T*-values <0 were downregulated (Boorsma et al., 2005). The *p*-value was corrected for multiple testing using the Benjamini–Hochberg method with *p*-adjusted < 0.05 assigned as significant (Benjamini and Hochberg, 1995).

### MRM-hr

Proteins that were identified to be significantly different in iTRAQ experiments were confirmed using MRM-hr measurements as previously described (Luu et al., 2018). Two or three tryptic peptides from each protein were chosen (Supplementary Table S2) and precursor m/z entered into MRM-hr methods. Each peptide selected was based on the following criteria: 8–20 amino acids in length, no missed cleavage, and no potential ragged ends or contained amino acids susceptible to variable modifications such as cysteine and methionine. “Control proteins” for normalization were also chosen based on the iTRAQ data with additional criteria: FC of 1 ± 0.1 and a coefficient of variation (CV) <10%.

For MRM-hr runs, 10 μg of proteins were digested as described in Luu et al. (2017) and loaded on the LC/TripleTOF 5600+ (SCIEX) and peptides separated over a 30-min gradient. The resulting data were imported into Skyline (v3.5.0.9319) (MacLean et al., 2010); then, intensity measurements from four to six transitions were determined. The inbuilt Skyline R-package, MS-stats (Choi et al., 2014), was then used to determine significantly differentially expressed proteins with linear mixed-effect models (Chang et al., 2012).

## Results

### *In vitro* Growth Measurements and Confirmation of 5 mM MgSO_4_-Induced Bvgi Phenotype

After 12 h incubation in THIJS with 5 mM MgSO_4_, the CFU/ml were recorded for L1423 and L1191. The CFU/ml for L1423 was 3.17 × 10^9^ ± 2.89 × 10^9^ CFU/ml while L1191 had 2.27 × 10^9^ ± 1.32 × 10^9^ CFU/ml with the difference being insignificant using a two-tailed Student’s *t*-test (*p* = 0.51).

To confirm the induction of the Bvgi phenotype with 5 mM MgSO_4_, the relative mRNA expression of *bipA* was quantified between L1423 and L1191 grown in THIJS with and without 5 mM MgSO_4_. *bipA* was selected as it is known to be optimally expressed in the Bvgi phenotype (Stockbauer et al., 2001; de Gouw et al., 2014b). The expression of *bipA* was found to be significantly upregulated when L1191 (FC = 5, *p* = 0.03) and L1423 (FC = 1.54, *p* = 0.04) were grown in the presence of 5 mM MgSO_4_, therefore confirming the induction of Bvgi phenotype (Supplementary Figure S1).

### Comparison of Whole Cell Proteome Under Intermediate Modulation

**FIGURE 2:**
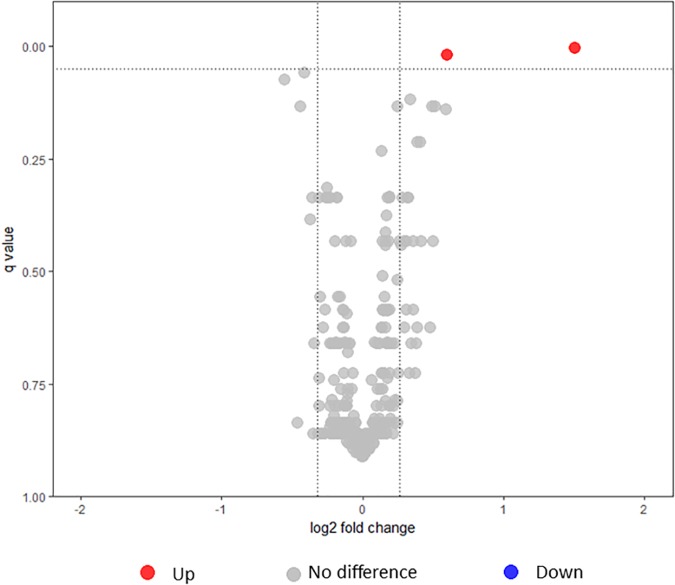
Volcano plot illustrating the dispersion of proteins identified in the whole cell under intermediate modulating conditions using iTRAQ. Dotted lines indicate fold change and false discovery rate cutoff. Red circles depict proteins that were significantly upregulated in L1423, and gray circles depict proteins that showed no differences. No proteins were found to be significantly downregulated.

From the whole cell, a total of 690 proteins were identified (20% of the total *B. pertussis* proteome) when *B. pertussis* was grown under intermediate modulating conditions (5 mM MgSO_4_). In the first iTRAQ, 603 proteins were identified while in the second iTRAQ, 601 proteins were identified (Supplementary Table S3). There were 513 proteins that were commonly identified, as shown in Figure 2 and Supplementary Table S3, and these proteins were used for quantitative analysis between L1423 and L1191. The functional categories and cellular locations for 690 proteins identified under intermediate modulating condition (5 mM MgSO4) were compared with the 825 proteins previously identified under non-modulating condition (Luu et al., 2018). There was no significant difference in the proportion of functional categories or cellular locations of proteins identified between both conditions (Supplementary Table S4). Furthermore, the proportion of immunogenic (*p* = 0.08) and Bvg-regulated proteins (*p* = 0.95) also showed no difference between the two conditions with Fisher’s exact test.

The expression of proteins in the whole cell between L1423 and L1191 under intermediate modulating conditions were compared using iTRAQ. BP0200, a tripartite tricarboxylate transporter (TCTC) protein, and BP1175, a hypothetical protein with a bacterial oligosaccharide-binding fold (BOF) domain, were found to be significantly upregulated in L1423 (Table 1). Both proteins were also previously identified to be upregulated in L1423 in non-modulating conditions (Luu et al., 2018). No proteins were significantly downregulated.

**Table 1:** Differentially expressed proteins identified by iTRAQ in the whole cell and secretome of L1423 when 5 mM MgSO_4_ is added.

Sub-proteome level	Locus	Gene	Product	Functional category	Fold change (L1423/L1191)	*T*-test (*p* ≤ 0.05)	*q*-value (*q* ≤ 0.05)
Whole cell	BP1175^#^		Hypothetical protein	Cell surface	1.52	0.00008	0.02
	BP0200^#^	*tctC*	Tripartite tricarboxylate transporter family receptor	Cell surface	2.84	0.00001	0.005
Secretome	BP2256^$^	*bsp22*	Type III secretion tip protein	Pathogenicity	0.48	0.004	0.02
	BP2253^$^	*bopD*	Type III secretion system outer protein D	Pathogenicity	0.56	0.003	0.016
	BP500^$^	*bteA*	Type III secretion toxin, effector	Pathogenicity	0.56	0.005	0.02
	BP2257^$^	*bopN*	Type III secretion outer protein N, effector	Pathogenicity	0.58	0.002	0.01
	BP1906^∗^	*ppiB*	Peptidyl-prolyl *cis*-trans isomerase B	Cell processes	1.21	0.001	0.009
	BP2540^∗^	*sucD*	Succinyl-coA synthetase subunit alpha	Energy metabolism	1.22	0.0002	0.004
	BP1770^∗^	*cspA*	Cold shock-like protein	Adaptation	1.23	0.003	0.016
	BP1054^∗^	*prn*	Pertactin	Pathogenicity	1.28	0.0002	0.004
	BP1201^$^	*tcfA*	Tracheal colonization factor	Pathogenicity	1.66	0.001	0.009
	BP0200^$^	*tctC*	Tripartite tricarboxylate transporter family receptor	Cell surface	2.52	<0.00001	<0.00001

*^#^Proteins that were not tested in MRM-hr. ^$^Proteins that were confirmed to be significantly different using MRM-hr. ^∗^Proteins that were unable to be confirmed using MRM-hr.*

When *q* value was assessed at 0.1 for virulence proteins that showed a trend in differential expression, Btc22, a T3SS chaperone protein was downregulated in L1423 (FC = 0.75, *q* = 0.06). At *q* < 0.2, BcrH1, another T3SS chaperone protein was also downregulated (FC = 0.74, *q* = 0.13) while BipA showed a trend in upregulation in L1423 compared to L1191 (FC = 1.4, *q* = 0.13).

**FIGURE 3:**
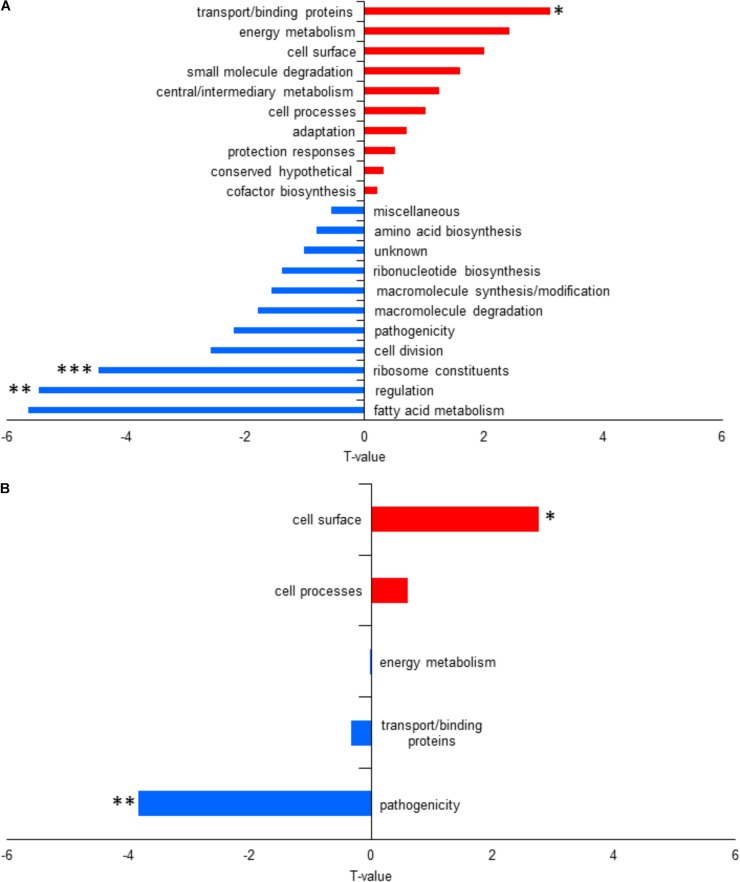
Functional category enrichment analysis using T-profiler of **(A)** the whole cell and **(B)** the secretome under intermediate modulating conditions. Positive *T*-values in red signifies upregulated functional categories, while negative *T*-values in blue signifies downregulated functional categories. Significant difference with ^∗^*p* < 0.05, ^∗∗^*p* < 0.005, and ^∗∗^*p* < 0.0005.

For functional enrichment analysis using T-profiler, three functional categories were significantly different. Regulation and ribosome constituents were significantly downregulated in L1423 with a *T*-value of -5.47 (*p*-adjust = 0.005) and -4.47 (*p*-adjust = 0.0005), respectively, while transport/binding proteins were significantly upregulated (*T*-value = 3.11 and *p*-adjust = 0.03) (Figure 3A and Supplementary Table S5).

### Comparison of Secretome Under Intermediate Modulation

**FIGURE 4:**
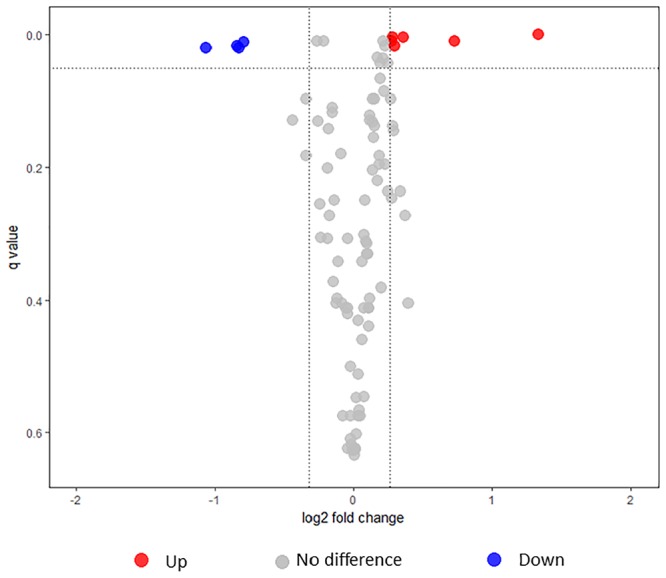
Volcano plot illustrating the dispersion of proteins identified in the secretome under intermediate modulating conditions using iTRAQ. Upregulated, downregulated, and non-significant proteins are shown as red, blue, and gray circles, respectively.

In the secretome, a total ofdownregulation under non-modulating 173 proteins were detected with 134 proteins identified in the first iTRAQ and 133 proteins in the second iTRAQ. Of these, 96 proteins were commonly identified and used for quantification analysis (Figure 4 and Supplementary Table S6). Of the 173 proteins, the secretion of 153 proteins could be accounted for bioinformatically and/or were detected in OMVs previously (Roberts et al., 2008; Raeven et al., 2015; Luu et al., 2017). Similar to that seen in the whole cell proteome, 173 proteins identified in 5 mM MgSO_4_ were compared to 225 secretome proteins identified in Luu et al. (2018) and there was no difference in the proportion of proteins from different cellular locations and functional categories between cells grown with and without 5 mM MgSO4 (Supplementary Table S7). Furthermore, the proportion of immunogenic proteins were marginally significant [Fisher’s exact test (*p* = 0.052)] but Bvg-regulated proteins [chi square test (*p* = 0.52)] had no significant differences (Supplementary Table S7).

Under intermediate modulating conditions, 10 proteins were significantly different between L1423 and L1191 (Table 1). Four proteins (Bsp22, BteA, BopD, and BopN) were significantly downregulated in L1423; all of which belonged to the T3SS. Bsp22 was also previously identified to be significantly downregulated in the secretome of L1423 under non-modulating conditions while BopD and BopN showed a trend of downregulation. Six proteins were significantly upregulated in L1423, two of which were virulence-associated (TcfA and Prn). TcfA was also previously identified to be upregulated in L1423 under non-modulated conditions while Prn was previously borderline significantly upregulated (FC = 1.25, *q* = 0.053) (Luu et al., 2018). The other four proteins were functionally associated with cell processes (PpiB), energy metabolism (SucD), adaptation (CspA), and cell surface (BP0200). It is interesting to note that BP0200 has been found to be significantly upregulated in both the L1423 whole cell and secretome under non-modulating and intermediate modulating conditions.

Expression differences of PtxB and Vag8 between L1423 and L1191 were found to be significant but their FC did not reach the cutoff for downregulation in L1423 at 0.8 (FC = 0.83–0.86 and *q* = 0.009). When FDR cut-off was analyzed at 0.1 and 0.2, sulphate-binding protein (Sbp) (FC 0.79, *q* = 0.1) and BipA (FC = 0.74, *q* = 0.13) were found to show a trend of downregulation in L1423. BipA also showed a trend in downregulation under non-modulating conditions (Luu et al., 2018). There was also an overall downregulation in L1423 of pathogenicity protein category with a *T*-value of -3.84 (*p*-adjust = 0.003) and an upregulation of cell surface proteins with a *T*-value of 2.77 (*p*-adjust = 0.02) (Figure 3B and Supplementary Table S5).

### MRM-hr Confirmation of Selected Proteins

For proteins that were identified to be differentially expressed by iTRAQ, MRM-hr was used as a confirmatory technique. Two proteins, BP0200 and BP1175, in the whole cell were increased under intermediate conditions. BP0200 was previously confirmed to be upregulated using MRM-hr in the whole cell and secretome of L1423 under non-modulating conditions while BP1175 was found to be deleted in the genome of L1191 (Luu et al., 2018). Therefore, MRM-hr was only performed for supernatant samples.

**FIGURE 5:**
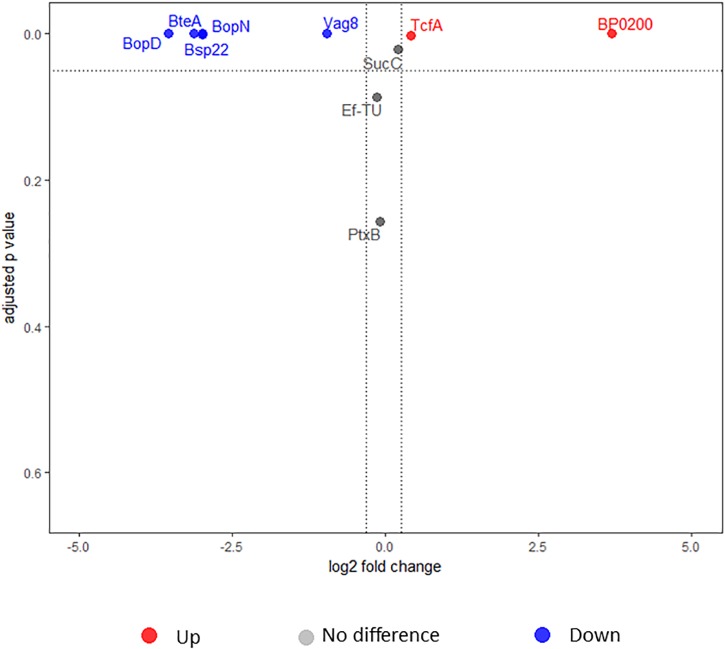
Volcano plot depicting secretome proteins tested using high-resolution multiple reaction monitoring. Red, blue, and gray circles represent upregulated, downregulated, and proteins that were not different, respectively.

Ten proteins were significantly different in the secretome when 5 mM MgSO_4_ was added. For four proteins (Prn, PpiB, SucD, and CspA), no suitable peptides for testing were identified. Hence, MRM-hr was only performed on 6 proteins (BteA, BopD, BopN, Bsp22, BP0200, and TcfA) to confirm expression differences. Furthermore, Vag8 and PtxB, which were significant but did not reach the FC cutoff for downregulation in the iTRAQ data were also tested. “Control proteins” for normalization were selected from the iTRAQ data and had the following criteria: FC 1 ± 0.1 and CV < 10%. From these criteria, SucC and Ef-Tu were chosen as control proteins. T3SS proteins including BteA, BopD, BopN, and Bsp22 were all confirmed to be significantly downregulated in L1423 by MRM-hr (FC = 0.09–0.12 and adjusted *p*-value <0.001) (Figure 5, Supplementary Figure S2, and Supplementary Table S8). Furthermore, Vag8 was also confirmed to be significantly downregulated in L1423 (FC = 0.51 and adjusted *p*-value < 0.001). MRM-hr also confirmed the upregulation of BP0200 (FC = 12 and adjusted *p*-value < 0.001) and TcfA in L1423 (FC = 1.33 and adjusted *p*-value = 0.002) (Figure 5, Supplementary Figure S2, and Supplementary Table S8). No significant difference was observed for PtxB (FC = 0.93 and adjusted *p*-value = 0.3) (Figure 5, Supplementary Figure S2, and Supplementary Table S8).

## Discussion

This study compared the whole cell proteome and secretome of L1423 and L1191, representative strains from SNP cluster I and SNP cluster II, respectively, and identified four proteins downregulated and seven proteins upregulated in L1423 under intermediate sulfate-modulating conditions. Sulfate is an important modulator for virulence factor expression and can be used to induce Bvgi (de Rossi et al., 2001; Friedman et al., 2001; de Gouw et al., 2014a,b,c), and we have confirmed that both of our strains were in the Bvgi state under the 5 mM MgSO_4_ modulation condition. The Bvgi intermediate phenotype in *B. pertussis* has been associated with transmission and biofilm formation (Irie et al., 2004; Vergara-Irigaray et al., 2005; de Gouw et al., 2014c). In this study, we found no significant differences in virulence factor expression in the whole cell proteome but differences in secreted virulence factors were found.

In the whole cell proteome, there were two proteins (BP0200 and BP1175) significantly different between L1423 and L1191 under intermediate modulating conditions. Both proteins were also identified in our previous study under non-modulating conditions to be significantly different in the whole cell, which confirms that regardless of the sulfate conditions, BP0200 and BP1175 are inherently differentially expressed between the two strains (Luu et al., 2018). BP0200 is a member of the tripartite TCTC family with a possible role in nutrient binding (Huvent et al., 2006). Our previous study identified an IS*481* element that was present upstream of BP0200 in L1191 but absent in L1423 (Table 2; Luu et al., 2018). It is possible that IS*481* insertion may affect the expression of BP0200.

**Table 2:** Summary of significantly differentially expressed proteins in non-modulating (Luu et al., 2018) and intermediate modulating conditions, and the potential reason for differential expression.

Protein	THIJS	THIJS + 5 mM MgSO_4_	Potential reason for differential expression
Type III secretion system effectors (BteA, Bsp22, BopD, and BopN)	Down	Down	T68C in *bscI* in L1423
Tracheal colonization factor	Up	Up	G527A in *tcfA* in L1191
BP1175	Up	Up	BP1175 deleted in L1191
BP0200	Up	Up	*IS*481 upstream of BP0200 in L1191

BP1175 contains an oligosaccharide-binding-fold (OB-fold) and is deleted in the genome of L1191 (Luu et al., 2018). Other proteins with this domain has been found in various pathogens, including *Salmonella* Typhimurium (VisP), *Vibrio cholerae* (VCA0732), and *Pseudomonas aeruginosa* (PA0320), to contribute to antimicrobial peptide and/or stress resistance (Fukushima et al., 2012; Moreira et al., 2013; Matson et al., 2017). In *S.* Typhimurium, VisP inhibits LpxO, an enzyme that modifies lipid A with the addition of a hydroxyl group, and results in increased resistance to antimicrobial peptides such as polymyxin B. Interestingly, *B. pertussis* also contains a LpxO homolog which suggests that BP1175 may also have a similar role in antimicrobial peptide and stress resistance (MacArthur et al., 2011). Genomic analysis of previously sequenced Australian *B. pertussis* strains also identified the deletion of BP1175 in 4 other strains belonging to SNP cluster II, V, and unclustered (Table 2; Bart et al., 2014; Safarchi et al., 2016b). This suggests that the observed difference in BP1175 are due to strain differences rather than cluster difference.

In the secretome, we identified five proteins including two adhesins that were significantly upregulated (TcfA, Prn, CspA, SucD, and PpiB). TcfA was previously found to be upregulated using iTRAQ in the whole cell and secretome of L1423 in unmodulated THIJS broth but could not be previously confirmed using MRM-hr (Luu et al., 2018). The upregulation of TcfA under intermediate modulating conditions was confirmed by MRM-hr (Table 2). CspA was also previously found under non-modulating conditions to be upregulated but only in the whole cell. Finally, in our previous study (Luu et al., 2018), Prn was nearly significantly upregulated in the secretome. This study was able to confirm the upregulation of Prn in L1423 under intermediate modulating conditions. Prn has been shown to be an important adhesin (Van Gent et al., 2011; Safarchi et al., 2015), and it is possible that the upregulation of Prn in L1423 may be associated with differences in Prn type. SNP cluster I strains contain Prn2 while SNP cluster II contain Prn3, and Tohama I, the ACV strain used in Australia contains Prn1 (Octavia et al., 2011). Prn1 contains five GGXXP amino acid repeats in region 1 (R1) while Prn2 contains six repeats in R1 as well as two amino acid differences (Mooi et al., 1998). These differences in the number of repeats were found to affect epitope recognition between antibodies generated against Prn1 and Prn2 (He et al., 2003). However, no difference was observed between Prn3 and Prn1 which contains the same number of amino acid repeats as Prn1 but differs in two amino acids (He et al., 2003). He et al. (2003) proposed that ACV antibodies generated against Prn1 can bind to Prn3 but may be less able to bind to Prn2, thus providing Prn2 strains with a selective advantage to avoid immune recognition. In addition to differences in epitope recognition, Prn1 confers greater colonization ability compared to Prn2 and Prn3, while no difference in colonization ability was observed between Prn2 and Prn3 strains (Van Gent et al., 2011). It is possible that a slight upregulation of Prn combined with Prn2 mismatch with ACV may allow L1423 to compensate for decreased colonization ability of Prn2 in hosts without adversely affecting immune recognition (Safarchi et al., 2016a).

The upregulation of Prn and TcfA, both of which are important adhesins, in our current and previous studies indicates the possibility that SNP cluster I may have increased adhesion and colonization ability compared to SNP cluster II. This hypothesis is supported by the mixed infection mice model results which demonstrated that SNP cluster I were better colonizers than cluster II regardless of the hosts immunization status (Safarchi et al., 2016a).

Several T3SS proteins were found to be significantly downregulated in this study including Bsp22, BopN, BopD, and BteA. Bsp22 forms the T3SS tip complex, while BopD is a translocase for the T3SS effectors BteA and BopN (Fennelly et al., 2008; Nagamatsu et al., 2009; Romero et al., 2013). Bsp22, BopN, and BopD were also previously found to be downregulated in the secretome in non-modulation conditions (Luu et al., 2018). However, previous comparative transcriptomic studies between *ptxP3* and *ptxP1* strains observed an increase in T3SS transcripts (King et al., 2013; de Gouw et al., 2014b). Moreover, a study comparing non-vaccine and vaccine type isolates in Japan also identified an increase in BteA while a similar study in French isolates identified no BteA expression differences between isolates (Han et al., 2011; Hegerle et al., 2013). Differences in the T3SS result between our study and others may be due to the culture time points used. For example, Han et al. (2011) showed that BteA expression peaks at 24 h, while our proteomic study was conducted at 12 h. It is possible that at an alternative time point, the relative expression of the T3SS between strains may be different. The expression of the T3SS can also be affected by the number of *in vitro* passages (Gaillard et al., 2011). However, since L1423 and L1191 were cultured in parallel, it is unlikely that reduced T3SS secretion in L1423 is solely due to *in vitro* passaging.

We examined the T3SS genes for any genetic differences between the two strains used to explain the downregulation of T3SS proteins. No mutations were found within the genes or promoters of the four T3SS genes, *bsp22*, *bopD*, *bopN*, or *bteA*. In L1423, a non-synonymous SNP was found in *bscI* at nucleotide position 68 (Table 2). *bscI* encodes a T3SS basal body protein homologous to YscI in *Yersinia*. During the assembly of the T3SS in *Yersinia*, YscI forms the inner rod protein which allows YscF, the T3SS needle protein, to traverse through the inner membrane (Dewoody et al., 2013). YscI was also found to bind to YscF for assembly of the hollow needle required for the secretion of other T3SS effectors (Cao et al., 2017). The non-synonymous SNP in *bscI* changed the amino acid from tyrosine to cysteine and was predicted by PROVEAN (v1.1.3) to have a deleterious effect (PROVEAN score = -7.2) on protein function (Choi and Chan, 2015). This suggests that BscI may be impaired in L1423, possibly affecting needle formation for effective secretion of T3SS proteins such as Bsp22, BopD, BopN, and BteA. This hypothesis is supported by studies in *Yersinia*, where single amino acid mutations to YscI which impaired binding to YscF, was found to reduce the secretion of YopN and LcrV, homologs to BopN and Bsp22, respectively (Cao et al., 2017). Interestingly, genomic analysis has found the same mutation to be present in all SNP cluster I strains as well as other global *ptxP3* strains (Bart et al., 2014; Sealey et al., 2014; Safarchi et al., 2016b). Therefore, it is possible that decreased secretion of T3SS effectors may be common across other global *ptxP3* strains and may be advantageous in reducing immune recognition to allow current strains to remain hidden from the immune system. Previous *in vivo* studies have identified Bsp22 and BteA from *B. bronchiseptica* to be highly immunogenic and protective (Medhekar et al., 2009); however, the homologous proteins in *B. pertussis* were not identified to be immunogenic or protective *in vivo* (Hegerle et al., 2013; Romero et al., 2013). Romero et al. (2013) proposed that the inability of T3SS effectors to be protective *in vivo* may be due to decreased expression or secretion of T3SS effectors in *B. pertussis* compared to *B. bronchiseptica*. A western blot by Ahuja et al. (2016) comparing Bsp22 expression in RB50, a *B. bronchiseptica* strain, with a Tohama I-derived strain and two other Californian clinical *B. pertussis* strain appears to support decreased expression of Bsp22 in *B. pertussis*; however, further work is required to confirm this. It is plausible that decreased secretion of T3SS proteins in SNP cluster I may be an ongoing phenomenon to reduce immune recognition in the evolution of *B. pertussis* from its ancestor, *B. bronchiseptica*.

In addition to the T3SS proteins, three virulence proteins showed a trend of downregulation (BipA, Vag8, and PtxB) in the supernatant using iTRAQ. Downregulation of PtxB was not confirmed but Vag8 downregulation was confirmed using MRM-hr. A trend of downregulation in BipA was also previously found in the supernatant of L1423 under non-modulating conditions and was confirmed using MRM-hr (Luu et al., 2018). Both Vag8 and BipA are highly immunogenic proteins with BipA and Vag8 in mice have been shown to be protective in previous immunization studies (de Gouw et al., 2014a,c). Similar to the T3SS, it is possible that the downregulation of BipA and Vag8 may also allow L1423 to lower host immune recognition and contribute to the increased fitness observed in the mouse model (Safarchi et al., 2016a).

Our study did not identify any differences in the expression of sulfatase-containing proteins or sulfate transporters except for a slight but non-significant downregulation of sulfate-binding protein (Sbp) in L1423 (FC = 0.79 and *q* = 0.1). By contrast, de Gouw et al. (2014b) found that Dutch *ptxP3* strains had decreased expression of sulfatase-containing protein genes and increased expression of sulfate transporter genes, such as *sbp*. It is possible that the differences observed between the two studies may be due to the difference in time points or strains used, or post-transcriptional modifications (Bibova et al., 2013, 2015). Furthermore, the majority of differences seen in this study were observed in the secretome which may not be detectable in transcriptomic experiments if the differences are due to protein secretion and not expression. However, it should be noted that in a recent *in vivo* transcriptomic study by van Beek et al. (2018), no differences in susceptibility to sulfate modulation response were observed during infection between their *ptxP3* and *ptxP1* strains, therefore supporting the results in this study.

In this study, we only examined Bvgi condition as 5 mM MgSO_4_ induced the Bvgi phenotype in both L1423 and L1191. The Bvg- condition should be investigated in future studies. Recently, RNA sequencing has uncovered hundreds of additional Bvg- genes that are involved in metabolism including sugar and amino acid transport, lipid metabolism, glyoxylate cycle, and phenylacetic acid degradation (Moon et al., 2017). Some of these Bvg- genes were identified to be expressed during *in vivo* infection, which suggests that the Bvg- phase may play a role in survival, transmission, and persistence (van Beek et al., 2018). Additionally, examining a wider spectrum of modulations such as other MgSO_4_ concentrations and additional modulators such as nicotinic acid as well as conditions that mimic the *in vivo* environment such as low glutamate, iron, and oxygen/carbon dioxide availability may also elucidate more phenotypic differences that contribute to the predominance of SNP cluster I.

## Conclusion

Whole cell proteome and secretome responses of Australian strains from SNP cluster I (*ptxP3*) and SNP cluster II (*ptxP1*) to intermediate levels of sulfate appear to be similar. Although sulfate is an important modulator for *B. pertussis* virulence gene expression, based on this study, our findings indicate that it is not a major factor for pathogen adaptation and the predominance of SNP cluster I strains in Australia. Of the 11 proteins identified to be significantly different between L1423 and L1191 under intermediate modulating conditions, nine were also differentially expressed in our previous proteomic study under non-modulated conditions (Luu et al., 2018). These include the upregulation of BP0200, CspA, TcfA, and Prn and the downregulation of T3SS and BipA. Notably, the upregulation of Prn and TcfA in SNP cluster I may result in increased colonization ability while the downregulation of the T3SS and other immunogenic proteins may reduce immune recognition, therefore leading to increased fitness of *B. pertussis*.

## Author Contributions

LL, SO, and RL designed the study. LL performed the proteomics experiments and drafted the manuscript. LL, SO, LZ, MR, and RL analyzed the results. SO, LZ, MR, VS, and RL provided critical revision of manuscript.

## Conflict of Interest Statement

The authors declare that the research was conducted in the absence of any commercial or financial relationships that could be construed as a potential conflict of interest.

## Supplementary Material

FIGURE S1 RT-qPCR confirmation of Bvgi induction when 5 mM MgSO_4_ was added. Relative fold change of *bipA* in L1191 and L1423 grown with 5 mM MgSO_4_ were compared to L1191 and L1423 grown without MgSO_4_, respectively. Error bars displays the 95% confidence intervals, Significant difference with ^∗^*p* < 0.05.

FIGURE S2 Floating bar graph of MRM-hr result for each protein selected. Ef-Tu and SucC were selected as control proteins to normalize against run variation between each sample. The relative abundance of each selected protein in L1191 and L1423 were analyzed with MRM-hr. The thick horizontal line across each bar represents the mean log2 intensity value for the selected protein and was calculated by averaging the mean log2 intensity of each tryptic peptide selected [shown as colored (green, red, and blue) symbols].

TABLE S1 List of primers used for RT-qPCR.

TABLE S2 MRM-hr peptides chosen for confirmation of proteins differentially expressed in the secretome between L1423 and L1191.

TABLE S3 Proteins identified in the whole cell of L1423 and L1191 using iTRAQ. Two sets of seven-plex iTRAQ were performed with three biological replicates in each set. The value shown in columns I–T is the fold change ratio for each biological replicate relative to the pooled reference. The ratios were then averaged for the six biological replicates and divided to obtain the fold change value of L1423 relative to L1191. The accession number, gene and product name, functional category, cellular localization, and whether the proteins are Bvg regulated or previously identified to be immunogenic are also shown.

TABLE S4 Comparison of the total number of whole cell proteins identified between THIJS and THIJS + 5 mM MgSO_4_ in each functional category, cellular localization, immunogenicity, and Bvg regulation. The Fisher’s exact or chi square test *p*-value is also shown.

TABLE S5 *T*-values and *p*-values for functional category enrichment analysis using T-profiler of the whole cell and the secretome under intermediate modulating conditions.

TABLE S6 Proteins identified in the L1423 and L1191 secretome using iTRAQ. The value shown in columns K–V is the fold change ratio for each biological replicate relative to the pooled reference. The ratios were then averaged for the six biological replicates and divided to obtain the fold change value of L1423 relative to L1191. The accession number, gene and product name, functional category, cellular localization, and whether the proteins are Bvg regulated, predicted to be secreted, or previously identified to be immunogenic are also shown.

TABLE S7 Comparison of the total number of secretome proteins identified between THIJS and THIJS + 5 mM MgSO_4_ in each functional category, cellular localization, immunogenicity, and Bvg regulation. The Fisher’s exact or chi square test *p*-value is also shown.

TABLE S8 Fold change and adjusted *p*-value for MRM-hr confirmation of differentially expressed proteins in the secretome.

## Abbreviations

ACV: acellular vaccine
FC: fold change
FDR: false discovery rate
iTRAQ: isobaric tag for relative and absolute quantitation
MRM-hr: high-resolution multiple reaction monitoring
OMV: outer membrane vesicles
RT-qPCR: real-time quantitative PCR
SNP: single-nucleotide polymorphism
T3SS: type III secretion system
THIJS: Thalen–IJssel
WCV: whole cell vaccine

## References

[B1] Ahuja U. Shokeen B. Cheng N. Cho Y. Blum C. Coppola G. (2016). Differential regulation of type III secretion and virulence genes in *Bordetella pertussis* and *Bordetella bronchiseptica* by a secreted anti-σ factor. *Proc. Natl. Acad. Sci. U.S.A.* 113 2341–2348. 10.1073/pnas.1600320113 26884180 PMC4780644

[B2] Bart M. J. Harris S. R. Advani A. Arakawa Y. Bottero D. Bouchez V. (2014). Global population structure and evolution of *Bordetella pertussis* and their relationship with vaccination. *mBio* 5:e01074-14. 10.1128/mBio.01074-14 24757216 PMC3994516

[B3] Bart M. J. van Gent M. van der Heide H. G. Boekhorst J. Hermans P. Parkhill J. (2010). Comparative genomics of prevaccination and modern *Bordetella pertussis* strains. *BMC Genomics* 11:627. 10.1186/1471-2164-11-627 21070624 PMC3018138

[B4] Benjamini Y. Hochberg Y. (1995). Controlling the false discovery rate: a practical and powerful approach to multiple testing. *J. R. Stat. Soc. B* 57 289–300.

[B5] Bhagwat A. A. Phadke R. P. Wheeler D. Kalantre S. Gudipati M. Bhagwat M. (2003). Computational methods and evaluation of RNA stabilization reagents for genome-wide expression studies. *J. Microbiol. Methods* 55 399–409. 10.1016/S0167-7012(03)00175-1 14529961

[B6] Bibova I. Hot D. Keidel K. Amman F. Slupek S. Cerny O. (2015). Transcriptional profiling of *Bordetella pertussis* reveals requirement of RNA chaperone Hfq for Type III secretion system functionality. *RNA Biol.* 12 175–185. 10.1080/15476286.2015.1017237 25674816 PMC4615762

[B7] Bibova I. Skopova K. Masin J. Cerny O. Hot D. Sebo P. (2013). The RNA chaperone Hfq is required for virulence of *Bordetella pertussis*. *Infect. Immun.* 81 4081–4090. 10.1128/IAI.00345-13 23980112 PMC3811842

[B8] Boorsma A. Foat B. C. Vis D. Klis F. Bussemaker H. J. (2005). T-profiler: scoring the activity of predefined groups of genes using gene expression data. *Nucleic Acids Res.* 33(Suppl. 2), W592–W595. 10.1093/nar/gki484 15980543 PMC1160244

[B9] Bowman J. P. Hages E. Nilsson R. E. Kocharunchitt C. Ross T. (2012). Investigation of the *Listeria monocytogenes* scott A acid tolerance response and associated physiological and phenotypic features via whole proteome analysis. *J. Proteome Res.* 11 2409–2426. 10.1021/pr201137c 22372944

[B10] Cao S.-Y. Liu W.-B. Tan Y.-F. Yang H.-Y. Zhang T.-T. Wang T. (2017). An interaction between the inner rod protein YscI and the needle protein YscF is required to assemble the needle structure of the yersinia type three secretion system. *J. Biol. Chem.* 292 5488–5498. 10.1074/jbc.M116.743591 28196868 PMC5392691

[B11] Chang C.-Y. Picotti P. Hüttenhain R. Heinzelmann-Schwarz V. Jovanovic M. Aebersold R. (2012). Protein significance analysis in selected reaction monitoring (SRM) measurements. *Mol. Cell. Proteomics* 11:M111.014662. 10.1038/nprot.2013.091 22190732 PMC3322573

[B12] Choi M. Chang C.-Y. Clough T. Broudy D. Killeen T. MacLean B. (2014). MSstats: an R package for statistical analysis of quantitative mass spectrometry-based proteomic experiments. *Bioinformatics* 30 2524–2526. 10.1093/bioinformatics/btu305 24794931

[B13] Choi Y. Chan A. P. (2015). PROVEAN web server: a tool to predict the functional effect of amino acid substitutions and indels. *Bioinformatics* 31 2745–2747. 10.1093/bioinformatics/btv195 25851949 PMC4528627

[B14] Coutte L. Huot L. Antoine R. Slupek S. Merkel T. J. Chen Q. (2016). The multifaceted RisA regulon of *Bordetella pertussis*. *Sci. Rep.* 6:32774. 10.1038/srep32774 27620673 PMC5020355

[B15] de Gouw D. de Jonge M. I. Hermans P. W. Wessels H. J. Zomer A. Berends A. (2014a). Proteomics-identified Bvg-activated autotransporters protect against *Bordetella pertussis* in a mouse model. *PLoS One* 9:e105011. 10.1371/journal.pone.0105011 25133400 PMC4136822

[B16] de Gouw D. Hermans P. W. Bootsma H. J. Zomer A. Heuvelman K. Diavatopoulos D. A. (2014b). Differentially expressed genes in *Bordetella pertussis* strains belonging to a lineage which recently spread globally. *PLoS One* 9:e84523. 10.1371/journal.pone.0084523 24416242 PMC3885589

[B17] de Gouw D. Serra D. O. de Jonge M. I. Hermans P. W. Wessels H. J. Zomer A. (2014c). The vaccine potential of *Bordetella pertussis* biofilm-derived membrane proteins. *Emerg. Microbes Infect.* 3:e58. 10.1038/emi.2014.58 26038752 PMC4150286

[B18] de Rossi B. N. P. Friedman L. E. Darnaud S. de Torres R. A. Franco M. A. (2001). Characterization of intermediate phenotypes induced by chemically undefined laboratory media in virulent *Bordetella bronchiseptica* strains. *J. Gen. Appl. Microbiol.* 47 39–46. 10.2323/jgam.47.39 12483567

[B19] Dewoody R. Merritt P. M. Marketon M. M. (2013). Regulation of the yersinia type III secretion system: traffic control. *Front. Cell. Infect. Microbiol.* 3:4. 10.3389/fcimb.2013.00004 23390616 PMC3565153

[B20] Dupré E. Herrou J. Lensink M. F. Wintjens R. Vagin A. Lebedev A. (2015). Virulence regulation with venus flytrap domains: structure and function of the periplasmic moiety of the sensor-kinase BvgS. *PLoS Pathog.* 11:e1004700. 10.1371/journal.ppat.1004700 25738876 PMC4352136

[B21] Dupré E. Wohlkonig A. Herrou J. Locht C. Jacob-Dubuisson F. Antoine R. (2013). Characterization of the PAS domain in the sensor-kinase BvgS: mechanical role in signal transmission. *BMC Microbiol.* 13:172. 10.1186/1471-2180-13-172 23883404 PMC3726324

[B22] Fennelly N. K. Sisti F. Higgins S. C. Ross P. J. van der Heide H. Mooi F. R. (2008). *Bordetella pertussis* expresses a functional type III secretion system that subverts protective innate and adaptive immune responses. *Infect. Immun.* 76 1257–1266. 10.1128/IAI.00836-07 18195025 PMC2258832

[B23] Friedman L. De Rossi B. P. Messina M. Franco M. (2001). Phenotype evaluation of *Bordetella bronchiseptica* cultures by urease activity and congo red affinity. *Lett. Appl. Microbiol.* 33 285–290. 10.1046/j.1472-765X.2001.00997.x 11559402

[B24] Fukushima K. Dubey S. K. Suzuki S. (2012). ygiW homologous gene from *Pseudomonas aeruginosa* 25W is responsible for tributyltin resistance. *J. Gen. Appl. Microbiol.* 58 283–289. 10.2323/jgam.58.283 22990488

[B25] Gaillard M. Bottero D. Castuma C. Basile L. Hozbor D. (2011). Laboratory adaptation of *Bordetella pertussis* is associated with the loss of type three secretion system functionality. *Infect. Immun.* 79 3677–3682. 10.1128/IAI.00136-11 21730086 PMC3165473

[B26] Han H.-J. Kuwae A. Abe A. Arakawa Y. Kamachi K. (2011). Differential expression of type III effector BteA protein due to IS481 insertion in *Bordetella pertussis*. *PLoS One* 6:e17797. 10.1371/journal.pone.0017797 21423776 PMC3053399

[B27] He Q. Mäkinen J. Berbers G. Mooi F. R. Viljanen M. K. Arvilommi H. (2003). *Bordetella pertussis* protein Pertactin induces type-specific antibodies: one possible explanation for the emergence of antigenic variants? *J. Infect. Dis.* 187 1200–1205. 10.1086/368412 12695998

[B28] Hegerle N. Rayat L. Dore G. Zidane N. Bedouelle H. Guiso N. (2013). In-vitro and in-vivo analysis of the production of the bordetella type three secretion system effector A in *Bordetella pertussis*, *Bordetella parapertussis* and *Bordetella bronchiseptica*. *Microbes Infect.* 15 399–408. 10.1016/j.micinf.2013.02.006 23470234

[B29] Hot D. Antoine R. Renauld-Mongenie G. Caro V. Hennuy B. Levillain E. (2003). Differential modulation of *Bordetella pertussis* virulence genes as evidenced by DNA microarray analysis. *Mol. Genet. Genomics* 269 475–486. 10.1007/s00438-003-0851-1 12768411

[B30] Huvent I. Belrhali H. Antoine R. Bompard C. Locht C. Jacob-Dubuisson F. (2006). Crystal structure of *Bordetella pertussis* BugD solute receptor unveils the basis of ligand binding in a new family of periplasmic binding proteins. *J. Mol. Biol.* 356 1014–1026. 10.1016/j.jmb.2005.11.096 16403514

[B31] Irie Y. Mattoo S. Yuk M. H. (2004). The Bvg virulence control system regulates biofilm formation in *Bordetella bronchiseptica*. *J. Bacteriol.* 186 5692–5698. 10.1128/JB.186.17.5692-5698.2004 15317773 PMC516841

[B32] King A. J. van der Lee S. Mohangoo A. van Gent M. van der Ark A. van de Waterbeemd B. (2013). Genome-wide gene expression analysis of *Bordetella pertussis* isolates associated with a resurgence in pertussis: elucidation of factors involved in the increased fitness of epidemic strains. *PLoS One* 8:e66150. 10.1371/journal.pone.0066150 23776625 PMC3679012

[B33] Kinnear S. M. Marques R. R. Carbonetti N. H. (2001). Differential regulation of Bvg-activated virulence factors plays a role in *Bordetella pertussis* pathogenicity. *Infect. Immun.* 69 1983–1993. 10.1128/IAI.69.4.1983-1993.2001 11254549 PMC98121

[B34] Kocharunchitt C. King T. Gobius K. Bowman J. P. Ross T. (2014). Global genome response of *Escherichia coli* O157: H7 sakai during dynamic changes in growth kinetics induced by an abrupt downshift in water activity. *PLoS One* 9:e90422. 10.1371/journal.pone.0090422 24594867 PMC3940904

[B35] Lacey B. (1960). Antigenic modulation of *Bordetella pertussis*. *Epidemiol. Infect.* 58 57–93. 10.1017/S0022172400038134 PMC2134314 14413273

[B36] Lesne E. Dupré E. Lensink M. F. Locht C. Antoine R. Jacob-Dubuisson F. (2018). Coiled-coil antagonism regulates activity of venus flytrap-domain-containing sensor kinases of the BvgS family. *mBio* 9:e02052-17. 10.1128/mBio.02052-17 29487240 PMC5829827

[B37] Lesne E. Dupré E. Locht C. Antoine R. Jacob-Dubuisson F. (2017). Conformational changes of inter-domain linker mediate mechanical signal transmission in sensor-kinase BvgS. *J. Bacteriol.* 199 e114–e117. 10.1128/JB.00114-17 28507245 PMC5573084

[B38] Lesne E. Krammer E.-M. Dupre E. Locht C. Lensink M. Antoine R. (2016). Balance between coiled-coil stability and dynamics regulates activity of BvgS sensor kinase in *Bordetella*. *mBio* 7:e02089-15. 10.1128/mBio.02089-15 26933056 PMC4810494

[B39] Luu L. D. W. Octavia S. Zhong L. Raftery M. Sintchenko V. Lan R. (2017). Characterisation of the *Bordetella pertussis* secretome under different media. *J. Proteomics* 158 43–51. 10.1016/j.jprot.2017.02.010 28242451

[B40] Luu L. D. W. Octavia S. Zhong L. Raftery M. Sintchenko V. Lan R. (2018). proteomic adaptation of Australian epidemic *Bordetella pertussis*. *Proteomics* 18:e1700237. 10.1002/pmic.201700237 29464899

[B41] MacArthur I. Jones J. Goodlett D. Ernst R. Preston A. (2011). Role of pagL and lpxO in *Bordetella bronchiseptica* lipid A biosynthesis. *J. Bacteriol.* 193 4726–4735. 10.1128/JB.01502-10 21764941 PMC3165656

[B42] MacLean B. Tomazela D. M. Shulman N. Chambers M. Finney G. L. Frewen B. (2010). Skyline: an open source document editor for creating and analyzing targeted proteomics experiments. *Bioinformatics* 26 966–968. 10.1093/bioinformatics/btq054 20147306 PMC2844992

[B43] Matson J. S. Livny J. DiRita V. J. (2017). A putative *Vibrio cholerae* two-component system controls a conserved periplasmic protein in response to the antimicrobial peptide polymyxin B. *PLoS One* 12:e0186199. 10.1371/journal.pone.0186199 29020117 PMC5636140

[B44] Medhekar B. Shrivastava R. Mattoo S. Gingery M. Miller J. F. (2009). Bordetella Bsp22 forms a filamentous type III secretion system tip complex and is immunoprotective in vitro and in vivo. *Mol. Microbiol.* 71 492–504. 10.1111/j.1365-2958.2008.06543.x 19040642 PMC2826148

[B45] Melvin J. A. Scheller E. V. Miller J. F. Cotter P. A. (2014). *Bordetella pertussis* pathogenesis: current and future challenges. *Nat. Rev. Microbiol.* 12 274–288. 10.1038/nrmicro3235 24608338 PMC4205565

[B46] Merkel T. J. Stibitz S. Keith J. M. Leef M. Shahin R. (1998). Contribution of regulation by the bvg locus to respiratory infection of mice by *Bordetella pertussis*. *Infect. Immun.* 66 4367–4373. 9712789 10.1128/iai.66.9.4367-4373.1998 PMC108527

[B47] Mooi F. Van Der Maas N. De Melker H. (2014). Pertussis resurgence: waning immunity and pathogen adaptation–two sides of the same coin. *Epidemiol. Infect.* 142 685–694. 10.1017/S0950268813000071 23406868 PMC9151166

[B48] Mooi F. R. van Loo I. H. Van Gent M. He Q. Bart M. J. Heuvelman K. J. (2009). *Bordetella pertussis* strains with increased toxin production associated with pertussis resurgence. *Emerg. Infect. Dis.* 15 1206–1213. 10.3201/eid1508.081511 19751581 PMC2815961

[B49] Mooi F. R. Van Oirschot H. Heuvelman K. van der Heide H. G. Gaastra W. Willems R. J. (1998). Polymorphism in the *Bordetella pertussis* virulence factors P. 69/pertactin and pertussis toxin in the netherlands: temporal trends and evidence for vaccine-driven evolution. *Infect. Immun.* 66 670–675. 9453625 10.1128/iai.66.2.670-675.1998 PMC107955

[B50] Moon K. Bonocora R. P. Kim D. D. Chen Q. Wade J. T. Stibitz S. (2017). The BvgAS regulon of *Bordetella pertussis*. *mBio* 8:e01526-17. 10.1128/mBio.01526-17 29018122 PMC5635692

[B51] Moreira C. G. Herrera C. M. Needham B. D. Parker C. T. Libby S. J. Fang F. C. (2013). Virulence and stress-related periplasmic protein (VisP) in bacterial/host associations. *Proc. Natl. Acad. Sci. U.S.A.* 110 1470–1475. 10.1073/pnas.1215416110 23302685 PMC3557018

[B52] Nagamatsu K. Kuwae A. Konaka T. Nagai S. Yoshida S. Eguchi M. (2009). Bordetella evades the host immune system by inducing IL-10 through a type III effector, BopN. *J. Exp. Med.* 206 3073–3088. 10.1084/jem.20090494 20008527 PMC2806459

[B53] Nakamura M. M. Liew S.-Y. Cummings C. A. Brinig M. M. Dieterich C. Relman D. A. (2006). Growth phase-and nutrient limitation-associated transcript abundance regulation in *Bordetella pertussis*. *Infect. Immun.* 74 5537–5548. 10.1128/IAI.00781-06 16988229 PMC1594893

[B54] Octavia S. Maharjan R. P. Sintchenko V. Stevenson G. Reeves P. R. Gilbert G. L. (2011). Insight into evolution of *Bordetella pertussis* from comparative genomic analysis: evidence of vaccine-driven selection. *Mol. Biol. Evol.* 28 707–715. 10.1093/molbev/msq245 20833694

[B55] Octavia S. Sintchenko V. Gilbert G. L. Lawrence A. Keil A. D. Hogg G. (2012). Newly emerging clones of *Bordetella pertussis* carrying prn2 and ptxP3 alleles implicated in Australian pertussis epidemic in 2008–2010. *J. Infect. Dis.* 205 1220–1224. 10.1093/infdis/jis178 22416243

[B56] Prugnola A. Aricò B. Manetti R. Rappuoli R. Scarlato V. (1995). Response of the bvg regulon of *Bordetella pertussis* to different temperatures and short-term temperature shifts. *Microbiology* 141 2529–2534. 10.1099/13500872-141-10-2529 7582012

[B57] Raeven R. H. van der Maas L. Tilstra W. Uittenbogaard J. P. Bindels T. H. Kuipers B. (2015). Immunoproteomic profiling of *Bordetella pertussis* outer membrane vesicle vaccine reveals broad and balanced humoral immunogenicity. *J. Proteome Res.* 14 2929–2942. 10.1021/acs.jproteome.5b00258 25988566

[B58] Roberts R. Moreno G. Bottero D. Gaillard M. E. Fingermann M. Graieb A. (2008). Outer membrane vesicles as acellular vaccine against pertussis. *Vaccine* 26 4639–4646. 10.1016/j.vaccine.2008.07.004 18640169

[B59] Romero R. V. Bibova I. Cerny O. Vecerek B. Wald T. Benada O. (2013). The *Bordetella pertussis* type III secretion system tip complex protein Bsp22 is not a protective antigen and fails to elicit serum antibody responses during infection of humans and mice. *Infect. Immun.* 81 2761–2767. 10.1128/IAI.00353-13 23690400 PMC3719584

[B60] Royle J. Lambert S. B. (2015). Fifty years of immunisation in Australia (1964–2014): the increasing opportunity to prevent diseases. *J. Paediatr. Child Health* 51 16–20. 10.1111/jpc.12796 25586840

[B61] Ryan M. Murphy G. Ryan E. Nilsson L. Shackley F. Gothefors L. (1998). Distinct T-cell subtypes induced with whole cell and acellular pertussis vaccines in children. *Immunology* 93 1–10. 10.1046/j.1365-2567.1998.00401.x 9536112 PMC1364099

[B62] Safarchi A. Octavia S. Luu L. D. W. Tay C. Y. Sintchenko V. Wood N. (2015). Pertactin negative *Bordetella pertussis* demonstrates higher fitness under vaccine selection pressure in a mixed infection model. *Vaccine* 33 6277–6281. 10.1016/j.vaccine.2015.09.064 26432908

[B63] Safarchi A. Octavia S. Luu L. D. W. Tay C. Y. Sintchenko V. Wood N. (2016a). Better colonisation of newly emerged *Bordetella pertussis* in the co-infection mouse model study. *Vaccine* 34 3967–3971. 10.1016/j.vaccine.2016.06.052 27346304

[B64] Safarchi A. Octavia S. Wu S. Z. Kaur S. Sintchenko V. Gilbert G. L. (2016b). Genomic dissection of Australian *Bordetella pertussis* isolates from the 2008–2012 epidemic. *J. Infect.* 72 468–477. 10.1016/j.jinf.2016.01.005 26826518

[B65] Scarlato V. Arico B. Prugnola A. Rappuoli R. (1991). Sequential activation and environmental regulation of virulence genes in *Bordetella pertussis*. *EMBO J.* 10 3971–3975. 10.1002/j.1460-2075.1991.tb04967.x 1718746 PMC453138

[B66] Scarlato V. Prugnola A. Aricó B. Rappuoli R. (1990). Positive transcriptional feedback at the bvg locus controls expression of virulence factors in *Bordetella pertussis*. *Proc. Natl. Acad. Sci. U.S.A.* 87 6753–6757. 10.1073/pnas.87.17.6753 1697687 PMC54615

[B67] Schmittgen T. D. Livak K. J. (2008). Analyzing real-time PCR data by the comparative CT method. *Nat. Protoc.* 3 1101–1108. 10.1038/nprot.2008.73 18546601

[B68] Sealey K. L. Harris S. R. Fry N. K. Hurst L. D. Gorringe A. R. Parkhill J. (2014). Genomic analysis of isolates from the UK 2012 pertussis outbreak reveals that vaccine antigen genes are unusually fast evolving. *J. Infect. Dis.* 212 294–301. 10.1093/infdis/jiu665 25489002

[B69] Shilov I. V. Seymour S. L. Patel A. A. Loboda A. Tang W. H. Keating S. P. (2007). The paragon algorithm, a next generation search engine that uses sequence temperature values and feature probabilities to identify peptides from tandem mass spectra. *Mol. Cell. Proteomics* 6 1638–1655. 10.1074/mcp.T600050-MCP200 17533153

[B70] Smith A. M. Guzmán C. A. Walker M. J. (2001). The virulence factors of *Bordetella pertussis*: a matter of control. *FEMS Microbiol. Rev.* 25 309–333. 10.1111/j.1574-6976.2001.tb00580.x 11348687

[B71] Stefanelli P. Sanguinetti M. Fazio C. Posteraro B. Fadda G. Mastrantonio P. (2006). Differential in vitro expression of the brkA gene in *Bordetella pertussis* and *Bordetella parapertussis* clinical isolates. *J. Clin. Microbiol.* 44 3397–3400. 10.1128/JCM.00247-06 16954284 PMC1594684

[B72] Stockbauer K. E. Fuchslocher B. Miller J. F. Cotter P. A. (2001). Identification and characterization of BipA, a *Bordetella* Bvg-intermediate phase protein. *Mol. Microbiol.* 39 65–78. 10.1046/j.1365-2958.2001.02191.x 11123689

[B73] Storey J. D. Tibshirani R. (2003). Statistical significance for genomewide studies. *Proc. Natl. Acad. Sci. U.S.A.* 100 9440–9445. 10.1073/pnas.1530509100 12883005 PMC170937

[B74] Thalen M. van den IJssel J. Jiskoot W. Zomer B. Roholl P. de Gooijer C. (1999). Rational medium design for *Bordetella pertussis*: basic metabolism. *J. Biotechnol.* 75 147–159. 10.1016/S0168-1656(99)00155-8 10553654

[B75] van Beek L. F. de Gouw D. Eleveld M. J. Bootsma H. J. de Jonge M. I. Mooi F. R. (2018). Adaptation of *Bordetella pertussis* to the respiratory tract. *J. Infect. Dis.* 217 1987–1996. 10.1093/infdis/jiy125 29528444

[B76] Van Gent M. Van Loo I. H. Heuvelman K. J. De Neeling A. J. Teunis P. Mooi F. R. (2011). Studies on Prn variation in the mouse model and comparison with epidemiological data. *PLoS One* 6:e18014. 10.1371/journal.pone.0018014 21464955 PMC3064647

[B77] Veal-Carr W. L. Stibitz S. (2005). Demonstration of differential virulence gene promoter activation in vivo in *Bordetella pertussis* using RIVET. *Mol. Microbiol.* 55 788–798. 10.1111/j.1365-2958.2004.04418.x 15661004

[B78] Vergara-Irigaray N. Chávarri-Martínez A. Rodríguez-Cuesta J. Miller J. F. Cotter P. A. de Tejada G. M. (2005). Evaluation of the role of the Bvg intermediate phase in *Bordetella pertussis* during experimental respiratory infection. *Infect. Immun.* 73 748–760. 10.1128/IAI.73.2.748-760.2005 15664913 PMC547029

[B79] Warfel J. M. Zimmerman L. I. Merkel T. J. (2014). Acellular pertussis vaccines protect against disease but fail to prevent infection and transmission in a nonhuman primate model. *Proc. Natl. Acad. Sci. U.S.A.* 111 787–792. 10.1073/pnas.1314688110 24277828 PMC3896208

