# Supplementary figures and images for: Comparison of the Whole Cell Proteome and Secretome of Epidemic Bordetella pertussis Strains From the 2008–2012 Australian Epidemic Under Sulfate-Modulating Conditions
Source: Front Microbiol. 2018 Nov 27;9:2851. doi: 10.3389/fmicb.2018.02851 (PMC6277516; doi:10.3389/fmicb.2018.02851)

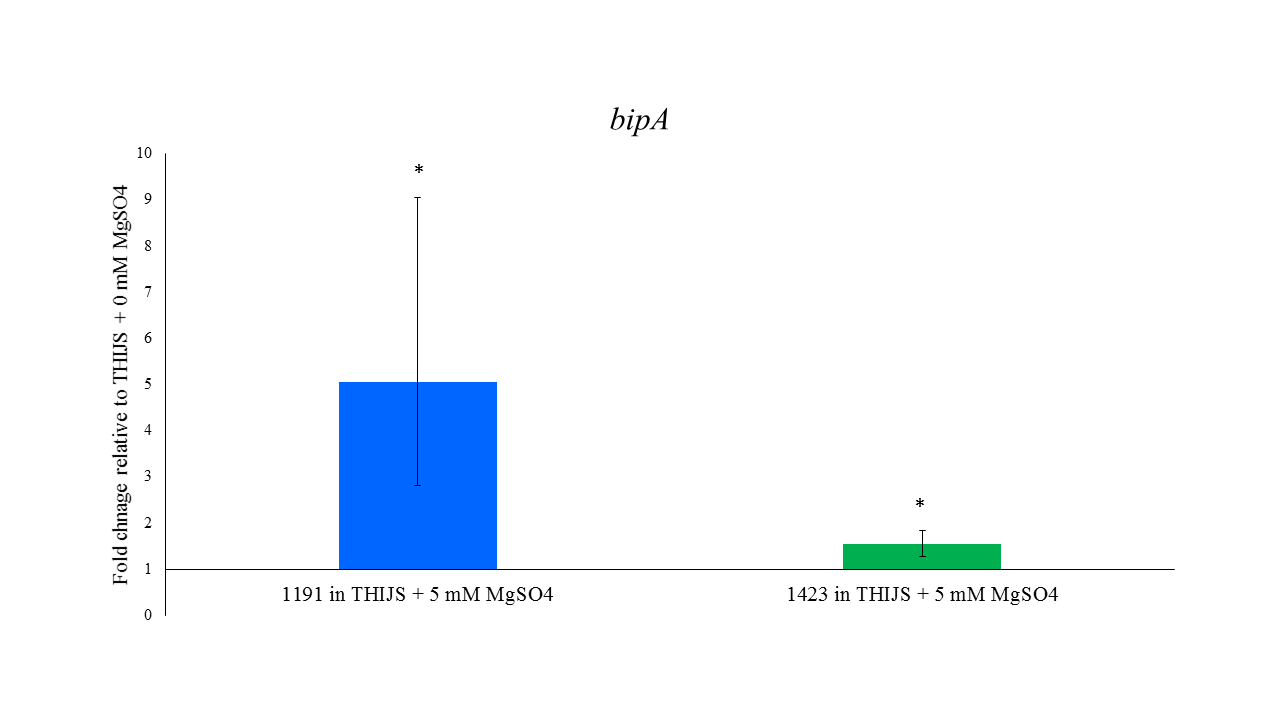

Supplement: FIGURE S1 — RT-qPCR confirmation of Bvgi induction when 5 mM MgSO4 was added. Relative fold change of bipA in L1191 and L1423 grown with 5 mM MgSO4 were compared to L1191 and L1423 grown without MgSO4, respectively. Error bars displays the 95% confidence intervals, Significant difference with ∗p < 0.05. [file Image_1.tif]

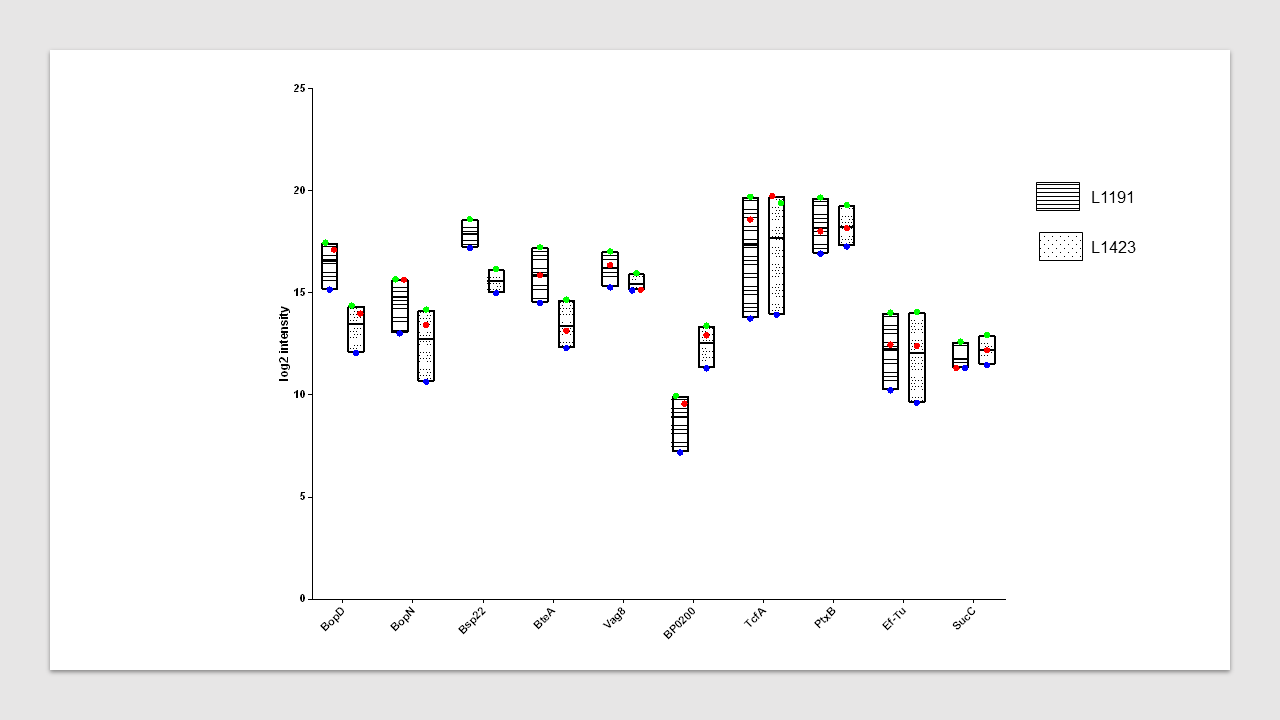

Supplement: FIGURE S2 — Floating bar graph of MRM-hr result for each protein selected. Ef-Tu and SucC were selected as control proteins to normalize against run variation between each sample. The relative abundance of each selected protein in L1191 and L1423 were analyzed with MRM-hr. The thick horizontal line across each bar represents the mean log2 intensity value for the selected protein and was calculated by averaging the mean log2 intensity of each tryptic peptide selected [shown as colored (green, red, and blue) symbols]. [file Image_2.TIF]
